# Timely Pulmonary Valve Replacement May Allow Preservation of Left Ventricular Circumferential Strain in Patients with Tetralogy of Fallot

**DOI:** 10.3389/fped.2017.00039

**Published:** 2017-02-28

**Authors:** Barbara E. U. Burkhardt, Marí Nieves Velasco Forte, Saravanan Durairaj, Isma Rafiq, Israel Valverde, Animesh Tandon, John Simpson, Tarique Hussain

**Affiliations:** ^1^Department of Pediatrics, University of Texas Southwestern Medical Center, Dallas, TX, USA; ^2^Pediatric Heart Center, University Children’s Hospital Zurich, Zurich, Switzerland; ^3^Division of Imaging Sciences and Biomedical Engineering, King’s College London, London, UK

**Keywords:** Tetralogy of Fallot, congenital heart defects, left ventricle, tissue tracking, strain, magnetic resonance, pulmonary valve replacement, myocardial function

## Abstract

**Introduction:**

Patients with Tetralogy of Fallot (TOF) and pulmonary insufficiency and a dilated right ventricle (RV) may suffer from a reduction in left ventricular (LV) performance. It is not clear whether timely pulmonary valve replacement (PVR) preserves LV mechanics.

**Methods:**

Ten TOF patients who underwent PVR were identified from hospital records, and pre- and postoperative cardiac magnetic resonance images were post-processed with a semi-automatic tissue tracking software. LV circumferential strain, time to peak strain, and torsion were compared before and after PVR. A control group of 10 age-matched normal volunteers was assessed as a comparison.

**Results:**

LV circumferential strain did not change before vs. after PVR (basal −18.3 ± 3.7 vs. −20.5 ± 3%, *p* = 0.082; mid-ventricular −18.4 ± 3.6 vs. −19.1 ± 2%, *p* = 0.571; apical −22.7 ± 5.2 vs. −22.1 ± 4%; *p* = 0.703). There was also no difference seen between the baseline strain and normal controls (control basal −18.2 ± 3.3%, *p* = 0.937; mid −18 ± 3.2%, *p* = 0.798; apex −24.1 ± 5%, *p* = 0.552). LV torsion remained unchanged from baseline to post PVR [systolic 2.75 (1.23–9.51) °/cm vs. 2.3 ± 1.2°/cm, *p* = 0.285; maximum 5.5 ± 3.5°/cm vs. 2.34 (1.37–8.07) °/cm, *p* = 0.083]. There was no difference in time to measured peak LV circumferential strain before vs. after PVR (basal 0.44 ± 0.1 vs. 0.43 ± 0.05, *p* = 0.912; mid-ventricular 0.42 ± 0.08 vs. 0.38 ± 0.06, *p* = 0.186; apical 0.40 ± 0.08 vs. 0.40 ± 0.06, *p* = 0.995). At the same time, pulmonary regurgitation and RV end-diastolic and end-systolic volume indices decreased and LV end-diastolic volume increased after PVR. RV and LV ejection fractions remained constant.

**Conclusion:**

PVR allows for favorable remodeling of both ventricular volumes for TOF patients with significant pulmonary regurgitation. In this cohort, LV myocardial functional parameters such as circumferential strain, time to peak strain, and LV torsion were normal at baseline and remain unchanged after PVR.

## Introduction

The right ventricle (RV) in patients with Tetralogy of Fallot (TOF) is at risk for progressive dilation secondary to pulmonary regurgitation. This may lead to an impairment of left ventricular (LV) mechanics, especially to a reduction in LV circumferential and radial strain, even in asymptomatic children and adolescents (1). Reduced RV longitudinal strain was shown to correlate with reduced LV longitudinal strain in adults with TOF (2), and LV circumferential and longitudinal strain have been associated with death or sustained ventricular tachycardia in TOF patients (3).

The indication for treatment by pulmonary valve replacement (PVR) is subject to much discussion currently, as both surgical and transcatheter PVR are being evaluated for their long-term benefit (4). The effects of PVR on myocardial mechanics have been described using echocardiography with tissue Doppler (5) and with speckle tracking or velocity vector analysis (6).

Cardiac magnetic resonance (CMR) is recommended in TOF patients for follow-up of RV volume and function (7). With the advent of tissue tracking, CMR cine images can be post-processed to measure biventricular strain and synchrony (8, 9).

Global circumferential strain has been shown to be the most reproducible measure of strain on CMR feature tracking (9). LV circumferential strain measured in a mid-ventricular slice by CMR feature tracking correlated with functional status and was one of the predictors of poor outcome in a large cohort study of 372 patients with TOF (10).

We hypothesized that LV circumferential strain and time to peak strain as well as LV torsion improve after PVR in TOF patients with dilated RV.

## Materials and Methods

### Patient Selection

The retrospective, anonymized use of data was approved by the St. Thomas’ Hospital Research Ethics Committee (London, England) 08/H0810/058. Written informed consent was not required.

Any patient who had undergone surgical PVR for pulmonary regurgitation after repair of TOF was included, if they had undergone pre- and post-procedure cine CMR including a short-axis cine stack of the left ventricle between July 2004 and August 2015, and the MRI study was available in the digital archive. Patients were excluded if other significant hemodynamic lesions were present (e.g., mitral regurgitation or significant branch pulmonary artery stenosis). Sixty-four surgical cases were performed in this time. Of these, only 14 met the inclusion criteria. Of these, four were excluded due to inadequate images.

Patients underwent surgical PVR 19 ± 9 years after initial tetralogy repair due to moderate to severe pulmonary regurgitation with significant RV dilation. The institutional policy at this time was for elective PVR if there was progressive dilation of the RV, significant RV diastolic chamber enlargement (>150 ml/m^2^), or any reduction in ventricular ejection fractions.

### Imaging

Cardiac magnetic resonance was performed on a 1.5 T scanner (Intera or Achieva, Philips Healthcare, Best, The Netherlands). ECG gated balanced cine steady-state free precession images were obtained in a short-axis stack of 9–13 slices from the atrioventricular valves to the apex, in 20–30 phases per cardiac cycle with a slice thickness of 8–10 mm, gap 0–2 mm, field of view between 272 mm × 272 mm and 390 mm × 390 mm, echo time 1.11–1.68 ms, temporal resolution at a median of 34.5 ms (25.3–50 ms), in breath-holding technique. CMR images of age-matched healthy volunteers were used as a control group.

Off-line post-processing was performed using cmr42 Release 5.3.4 (Circle Cardiovascular Imaging Inc., Calgary, AB, Canada). Ventricular volumes and ejection fractions of both ventricles were obtained from systolic and diastolic tracings as described elsewhere (11), and volumes were indexed to body surface area.

**Figure 1:**
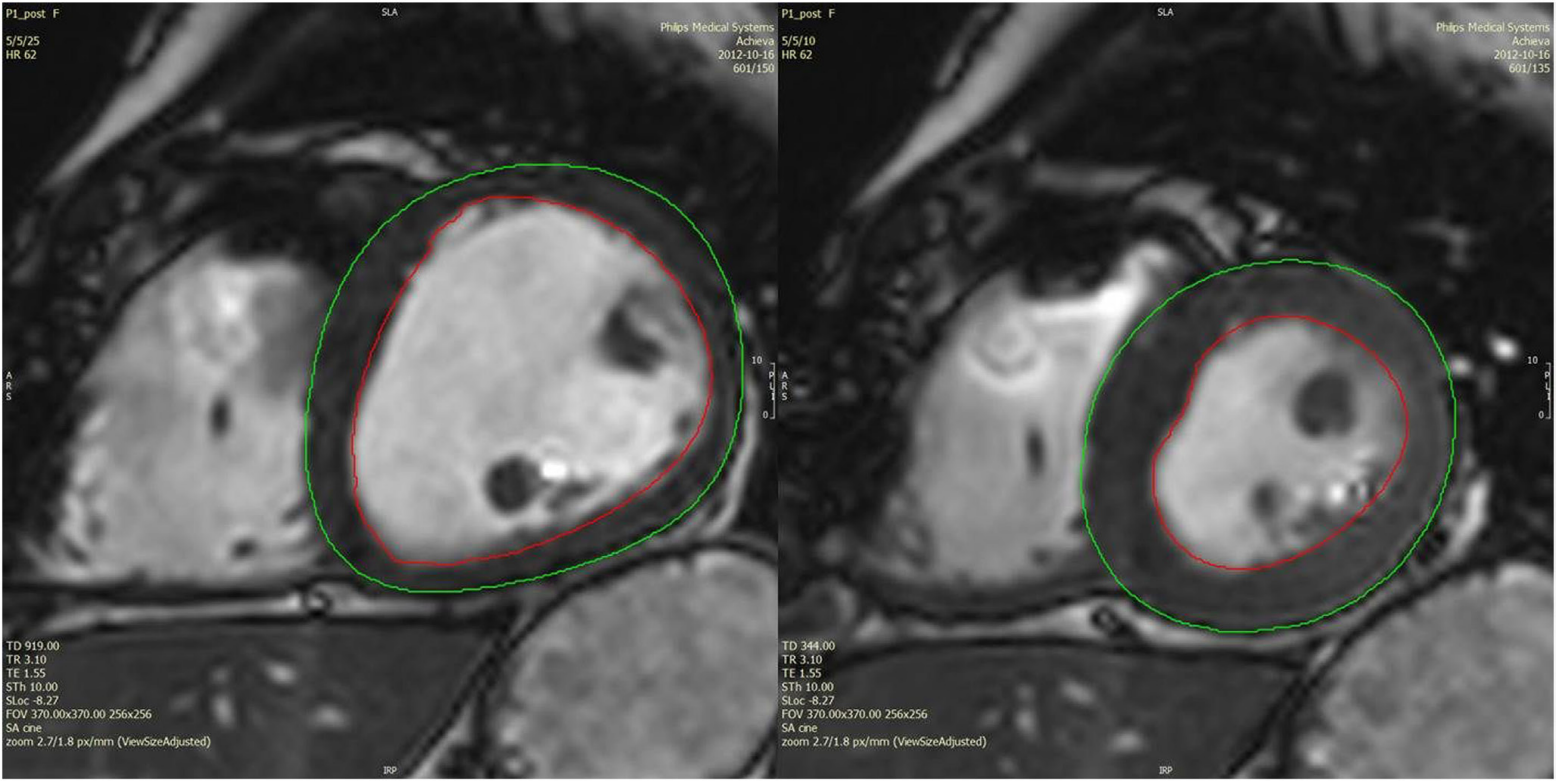
**Example of diastolic and systolic tracings of a mid-ventricular slice in a post-PVR patient**.

For tissue tracking analysis, basal, mid-ventricular, and apical slices were identified, which displayed myocardium along the entire LV circumference in all phases, avoiding the most basal and the most apical slices. End-diastole and end-systole were manually defined by comparing mid-ventricular slices in all phases. Endocardial and epicardial contours of the left ventricle were drawn manually, starting in end-diastole (Figure 1), tracked semi-automatically across all phases, and corrected manually where necessary, in order to accurately mark the endocardial and epicardial borders. Tissue tracking analysis (12) with a local heart coordinate system (13) was used to derive global circumferential strain curves of each slice as well as torsion of the LV. Maximal systolic values were used for analysis, only taking into account measured points, not interpolated graph data. Time to peak strain was measured as the number of phases from end-diastole to peak circumferential strain divided by the total number of phases, thus giving a measure independent of the patient’s heart rate.

### Statistical Methods

Ventricular volumes were indexed to body surface area. Statistical analyses were performed with IBM SPSS Statistics 24 (IBM Corporation, Armonk, NY, USA). Continuous data are expressed as mean ± SD or as median (range) as appropriate. The one-sample Kolmogorov–Smirnov test was used to test for normal distribution. Pre- and post-PVR conditions were compared by Wilcoxon signed-rank tests for non-normally distributed variables, and paired sample *t*-tests were used for normally distributed variables. Independent sample *t*-tests were used for comparisons between patient groups. Coefficients of variation (SDs of differences between two measurements, divided by the respective means of two measurements) and intraclass correlation coefficients were calculated to describe intra- and interobserver variability of measurements.

## Results

### Patient Characteristics

Ten patients qualified for pre- and postsurgical CMR analysis (seven males, three females). Patient and control subject ages, weights, and heart rates are summarized in Table 1.

**Table 1:** **Patient characteristics**.

	Before PVR	After PVR	Controls	*p*-Value before vs. after PVR	*p*-Value before PVR vs. controls
Age (years)	25.1 ± 10.5	29.1 ± 10.8	23.4 ± 3.7	0.002*	0.634
Weight (kg)	66.4 ± 13.3	68.6 ± 12.9	74.3 ± 19.4	0.565	0.301
Heart rate (bpm)	65.4 ± 10.9	63.8 ± 10.5	72.4 ± 11.8	0.660	0.184

*Statistical significance assumed for p < 0.05.

*PVR, pulmonary valve replacement*.

*Preoperative cardiac magnetic resonance (CMR) was performed 18 months prior to PVR (median; range 0–49 months). Postoperative CMR was performed 26 months after PVR (median; range 6–117 months)*.

### Ventricular Volumes and Function

Pulmonary regurgitation and RV end-diastolic and end-systolic volumes were significantly reduced after PVR compared to baseline. LV end-diastolic volume showed an increase, although it was not significantly abnormal before PVR. There were no changes in LV end-systolic volume, RV or LV ejection fractions (Table 2).

**Table 2:** **Ventricular volumes and function before and after PVR**.

	Before PVR	After PVR	Controls	*p*-Value before vs. after PVR	*p*-Value before PVR vs. controls
Pulmonary RF (%)	50.3 ± 12.9	12.4 ± 13.3	n/a	<0.001*	n/a
RVEDVi (ml/m^2^)	154 ± 22	111 ± 32	91 ± 23	0.006*	<0.001*
RVESVi (ml/m^2^)	79 ± 15	57 ± 19	41 ± 11	0.021*	<0.001*
RVEF (%)	49 ± 6	49 ± 6	55 ± 2	0.872	0.006*
LVEDVi (ml/m^2^)	78 ± 12 ml/m^2^	90 ± 20	89 ± 16	0.038*	0.107
LVESVi (ml/m^2^)	33 ± 7 ml/m^2^	37 ± 9	41 ± 8	0.142	0.025
LVEF (%)	57 ± 6	59 ± 6	54 ± 4	0.427	0.172

*Statistical significance assumed for p < 0.05.

*Values are expressed as mean ± SD*.

*PVR, pulmonary valve replacement; TOF, Tetralogy of Fallot; Pulmonary RF, pulmonary regurgitation fraction; RVEDVi, right ventricular end-diastolic volume indexed to body surface area; RVESVi, right ventricular end-systolic volume indexed to body surface area; RVEF, right ventricular ejection fraction; LVEDVi, left ventricular end-diastolic volume indexed to body surface area; LVESVi, left ventricular end-systolic volume indexed to body surface area; LVEF, left ventricular ejection fraction*.

### LV Circumferential Strain and Torsion

**Figure 2:**
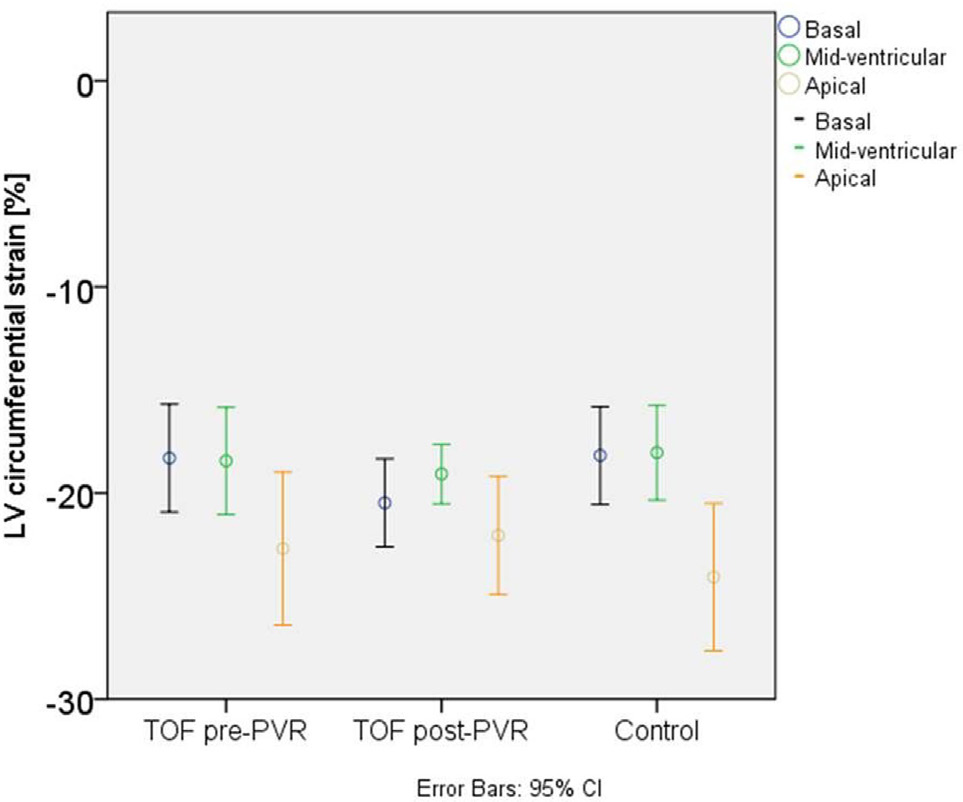
**Left ventricular (LV) circumferential strain in patients with Tetralogy of Fallot and in normal controls**.

Left ventricular circumferential strain or torsion did not change at the basal, the mid-ventricular, or the apical level after PVR compared to before PVR (Table 3; Figure 2). No difference in LV circumferential strain or torsion was seen between TOF patients at baseline and controls (Figure 2).

**Table 3:** **Left ventricular circumferential strain and torsion before and after PVR**.

	Before PVR	After PVR	Controls	*p*-Value before vs. after PVR	*p*-Value before PVR vs. controls
Basal LV circumferential strain (%)	−18.3 ± 3.7	−20.5 ± 3	−18.2 ± 3.3	0.082	0.937
Mid-ventricular LV circumferential strain (%)	−18.4 ± 3.6	−19.1 ± 2	−18 ± 3.2	0.571	0.798
Apical LV circumferential strain (%)	−22.7 ± 5.2	−22.1 ± 4	−24.1 ± 5	0.703	0.552
Systolic LV torsion (°/cm)	2.75 (1.23–9.51)	2.3 ± 1.2	4 ± 2.5	0.285	0.755

*Values are expressed as mean ± SD where appropriate, otherwise as median (range)*.

*PVR, pulmonary valve replacement; TOF, Tetralogy of Fallot; LV, left ventricle*.

### Time to Peak Circumferential Strain

The fraction of phases to peak LV circumferential strain based on the total number of phases per cardiac cycle did not differ before vs. after PVR (Table 4).

**Table 4:** **Proportion of phases to peak circumferential strain by total number of phases**.

	Before PVR	After PVR	Controls	*p*-Value before vs. after PVR	*p*-Value before PVR vs. controls
Basal LV	0.44 ± 0.1	0.43 ± 0.05	0.42 ± 0.08	0.912	0.721
Mid-ventricular LV	0.42 ± 0.08	0.38 ± 0.06	0.44 ± 0.1	0.186	0.729
Apical LV	0.40 ± 0.08	0.40 ± 0.06	0.44 ± 0.06	0.995	0.216

*Values are expressed as mean ± SD*.

*PVR, pulmonary valve replacement; TOF, Tetralogy of Fallot; LV, left ventricle*.

### Reproducibility

(a) Intraobserver variability results of 10 subjects (TOF *n* = 5; control *n* = 5) are presented in Table 5.
(b) Interobserver variability results of 10 subjects (TOF *n* = 5; control *n* = 5) are presented in Table 6.

**Table 5:** **Intraobserver variability**.

Intraobserver variability, *n* = 10						
	Mean value	Mean difference	SD of differences	Limits of agreement	CV (%)	ICC
Basal ε circ (%)	−18	1.36	2.61	−0.26; 3.0	14.2	0.790
Mid ε circ (%)	−16.7	0.28	3.00	−1.58; 2.13	13.7	0.681
Apical ε circ (%)	−22	−1.14	2.44	−2.65; 0.37	8.5	0.944
Basal phases to peak strain	0.40	−0.02	0.05	−0.06; 0.01	11.5	0.849
Mid phases to peak strain	0.42	−0.02	0.13	−0.10; 0.06	23.9	0.353
Apical phases to peak strain	0.40	0.01	0.02	−0.01; 0.02	5.5	0.961
Systolic LV torsion (°/cm)	3.43	1.42	2.96	−0.42; 3.25	66.8	0.250

*Basal ε circ, basal left ventricular circumferential strain; mid ε circ, mid left ventricular circumferential strain; apical ε circ, apical left ventricular circumferential strain; basal phases to peak strain, proportion of phases to peak basal left ventricular circumferential strain by total number of phases; mid phases to peak strain, proportion of phases to peak mid left ventricular circumferential strain by total number of phases; apical phases to peak strain, proportion of phases to peak apical left ventricular circumferential strain by total number of phases; LV, left ventricle; CV, coefficient of variation = SD of differences between two measurements, divided by mean of two measurements; ICC, intraclass correlation coefficient (average measures)*.

*Limits of agreement encompass the 95% confidence interval of the difference between measurements*.

**Table 6:** **Interobserver variability**.

	Mean value	Mean difference	SD of differences	Limits of agreement	CV (%)	ICC
Basal ε circ (%)	−17.8	0.77	1.28	−0.02; 1.57	5.6	0.942
Mid ε circ (%)	−16.3	−0.7	2.21	−2.07; 0.67	10.1	0.815
Apical ε circ (%)	−22.4	−0.44	2.83	−2.19; 1.32	6.1	0.922
Basal phases to peak strain	0.41	−0.05	0.08	−0.10; 0.00	17.9	0.263
Mid phases to peak strain	0.39	0.04	0.07	−0.01; 0.08	15.2	0.584
Apical phases to peak strain	0.40	0.01	0.04	−0.01; 0.03	7.7	0.902
Systolic LV torsion (°/cm)	3.67	0.92	2.48	−0.62; 2.46	41.8	0.621

*Basal ε circ, basal left ventricular circumferential strain; mid ε circ, mid left ventricular circumferential strain; apical ε circ, apical left ventricular circumferential strain; basal phases to peak strain, proportion of phases to peak basal left ventricular circumferential strain by total number of phases; mid phases to peak strain, proportion of phases to peak mid left ventricular circumferential strain by total number of phases; apical phases to peak strain, proportion of phases to peak apical left ventricular circumferential strain by total number of phases; LV, left ventricle; CV, coefficient of variation = SD of differences between two measurements, divided by mean of two measurements; ICC, intraclass correlation coefficient (average measures)*.

*Limits of agreement encompass the 95% confidence interval of the difference between measurements*.

## Discussion

Pulmonary valve replacement was effective in our patient cohort to reduce pulmonary regurgitation and RV end-diastolic and end-systolic volumes. LV end-diastolic volume increased after PVR, which has been shown by others before (14), and which is most likely due to the interventricular septum making way for LV filling after RV volume overload was relieved. RV and LV ejection fractions were not indicators of the improved physiology.

Interestingly, LV circumferential strain did not show a significant change before vs. after PVR in the basal, mid-ventricular, or apical slices either. Time to peak circumferential strain and LV torsion were also unchanged before vs. after PVR. Patients did not have different LV circumferential strain, LV torsion, or time to peak circumferential strain values than controls either before or after PVR.

Like many studies looking at the reproducibility of strain measurements (9, 12), our data also show very good intra- and inter-rater comparisons for LV circumferential strain at the basal, mid-ventricular, and apical levels. Time to peak strain agreement was highest in the apical slice. However, our LV torsion measurements were not as well reproducible using this tissue tracking technique.

### The Left Ventricle in Fallot Patients

We did not show any impairment in circumferential strain in our TOF patients compared to controls and no change after PVR. Furthermore, our study confirms the reproducibility of tissue tracking for LV strain analysis. However, the LV in TOF has been shown to suffer together with the RV in the long term. Indeed, adult patients with repaired TOF are presenting with an excess of early onset heart failure (15). This notion is supported by small animal models of chronic RV pressure loading, which show upregulation of LV fibrosis and apoptosis (16, 17). Recent work also suggests that serum biomarkers of heart failure are elevated in this group and track with the degree of right ventricular volume loading (18).

Therefore, it seems reasonable to presume that our findings are incongruent with these facts because our patients underwent early intervention which preserved their LV function. Normal values for LV circumferential strain in young adults by CMR feature tracking have been established in a large cohort by Augustine et al. (19), with LV circumferential strains of −22 ± 4% at the base, −18 ± 3% in mid-ventricular, and −21 ± 38% (*sic*) in apical slices. We found that our TOF patients had strain values largely in this range. Our findings are supported by Kempny et al., whose TOF patients had similar circumferential strain in a single mid-ventricular slice compared to their normal controls (20).

Although Padiyath et al. (21) reported reduced LV mid-ventricular circumferential strain in TOF patients compared to their normal volunteers (21), it should be noted that their TOF patients had strain values that were actually also within the normal range of the large healthy volunteer study (19). In keeping with this, in the largest analysis of strain in TOF patients performed to date (10), LV circumferential strain was also in the normal range [−21.6; 95% CI (18.9, 24.5)]. This finding is mirrored by findings from Moon et al. (3), again showing normal strain values in TOF patients (circumferential strain 23%) but reduced strain in a very small cohort that had adverse outcomes (17%, *p* = 0.003). A reduction in circumferential strain may therefore be a late sign of adverse LV myocardial condition.

### LV Torsion

Young adults with repaired TOF with and without PVR both show decreased LV twist on 3D echocardiography (22). There is very little available literature on LV torsion measured by CMR tissue tracking in patients with TOF. However, our data showed that reproducibility for LV torsion was poor, and others have shown considerable coefficients of variation in healthy volunteers before (12). The higher variability of torsion compared to circumferential strain is not surprising, because torsion is calculated from two separate LV slices instead of one. Further work is necessary before this analysis is applicable to these patients.

### Time to Peak Strain

Time to peak strain is easy to measure from strain curves and was prolonged for longitudinal strain in the RV of TOF patients in an echocardiographic speckle tracking analysis by Mueller et al. (23) but not in the LV [see also Ref. (24)]. However, as the LV primarily consists of circumferential fibers, the circumferential direction of deformation could be more indicative of dysfunctional LV mechanics, even more so in the context of a flattened interventricular septum in TOF patients with RV volume overload prior to PVR. The fact that our study did not find any difference in time to peak LV circumferential strain before and after PVR could be due to the relatively low number of phases per cardiac cycle, so that differences in short time intervals may have been missed.

### Pulmonary Valve Replacement

All patients in this study had PVR surgery. In a similarly small cohort of 13 patients with pulmonary regurgitation undergoing transcatheter PVR, most of them with an underlying diagnosis of TOF, Harrild et al. (25) found an increase in the amount of LV circumferential strain by CMR tissue tracking, even though mean values were higher than published normal (19) even prior to PVR.

In our cohort of patients with repaired TOF, LV myocardial deformation parameters were in normal ranges both before and after PVR. Again, this may be due to an institutional bias to replace a dysfunctional pulmonary valve early, before RV and LV function might suffer.

### Limitations

Patient recruitment for this analysis was retrospective. It is possible that statistical significance for some parameters was not reached because of the number of patients being too small. However, *post hoc* analysis showed that at a significance level of 0.05 and 80% power, the study was empowered to show a 3% difference in circumferential strain at the mid-ventricular level.

## Conclusion

Pulmonary valve replacement improves the interventricular interaction of TOF patients to volume unloading, but intrinsic LV myocardial function parameters such as LV circumferential strain and LV torsion, as measured by CMR tissue tracking, are normal in the majority of TOF patients and remain unchanged after PVR. The literature suggests that there is a small subgroup of patients that have reduced circumferential strain and have adverse outcomes, whereas torsion requires further study in this context.

## Ethics Statement

This study was carried out in accordance with the recommendations of the St. Thomas’ Hospital Research Ethics Committee (London, England) with a waiver of written informed consent from all subjects in accordance with the Declaration of Helsinki, for the retrospective use of anonymized data. The protocol was approved by the St. Thomas’ Hospital Research Ethics Committee (London, England) under 08/H0810/058.

## Author Contributions

BB contributed to research question, measured and analyzed data, interpreted data, and wrote the manuscript. MF contributed to research question, acquired data, and revised manuscript for intellectual content. SD and IR acquired data and revised manuscript for intellectual content. AT performed measurements and revised manuscript for intellectual content. JS revised manuscript for intellectual content. TH conceived research question, identified cases, analyzed data, interpreted data, and revised manuscript for intellectual content.

## Conflict of Interest Statement

The authors declare that the research was conducted in the absence of any commercial or financial relationships that could be construed as a potential conflict of interest.

## Abbreviations

CMR, cardiac magnetic resonance; LV, left ventricle; PVR, pulmonary valve replacement; RV, right ventricle; TOF, Tetralogy of Fallot.
